# Safety of electroconvulsive therapy in the context of physiological and medical complexity: A state‐of‐the art review

**DOI:** 10.1002/pcn5.70051

**Published:** 2025-01-03

**Authors:** Alby Elias, Soumitra Das, James Kirkland, Sarabjit Loyal, Naveen Thomas

**Affiliations:** ^1^ Department of Psychiatry The University of Melbourne Melbourne Victoria Australia; ^2^ Division of Mental Health and Wellbeing Western Health Melbourne Victoria Australia

**Keywords:** ECT, electroconvulsive therapy, medical complication, precaution, risk

## Abstract

Medical contraindications and complications pose challenges for electroconvulsive therapy (ECT). Most published reports are scattered across various physiological systems and individual disease conditions. This review aimed to evaluate the literature on physiological and medical complexities during ECT and discuss risk mitigation strategies in a comprehensive review. We searched PubMed and Embase for contraindications and precautions during ECT with relevant MeSH terms and appraised previous reviews on the same topic. The results suggest that mortality directly attributed to ECT is extremely rare. Instances of fatalities, including fetal deaths, have been reported after ECT in the presence of recent myocardial infarction, deep vein thrombosis, intracranial aneurysm and tumors, pheochromocytoma, sepsis, and pregnancy. However, there are no definite conclusions or consensus on attributions of the outcomes to ECT in all cases because of the time lag between the treatment and deaths and confounding factors. The risks can be mitigated with safety protocols, adequate stakeholder communication, collaboration with anesthetists and specialists, consultation‐liaison services, and ECT education. Overall, ECT remains a relatively safe treatment even in the presence of medical and physiological complexities. In rare instances, certain medical conditions may indicate a high risk for ECT, where practitioners avoid the treatment or administer it with precautions if the risk‐benefit ratio favors its use.

## INTRODUCTION

Electroconvulsive therapy (ECT), the oldest somatic intervention in psychiatry, continues to remain a highly effective treatment for mood and psychotic disorders.^1^, ^2^ Practitioners frequently encounter medical conditions that require precaution during ECT. Published reports have, however, formed a piecemeal literature with reports scattered across various physiological systems and individual disease conditions. This account collates the results into a single comprehensive review. Although the contraindications for ECT form well‐trodden ground, findings accrued since past reviews make it an evolving landscape.

ECT is an enigmatic treatment in medicine: it has an impressive safety record despite profound neurochemical and hemodynamic changes associated with seizures, such as a 15‐fold rise in adrenalin and up to two times increased blood pressure (BP).^3^, ^4^, ^5^ In contrast to the public fear of death from ECT, which was present in 20% of participants in one study, and a lay viewpoint that electric current is passed on to the brain, mortality directly related to ECT is extremely rare.^6^, ^7^, ^8^ The skull, with a relatively high impedance, attenuates the electric flow to the brain during ECT. Most of the current is short‐circuited around the skull.^9^ Therefore, the voltage gradient across a single neuron cell body drops to 0.006 V, far below the action potential required for depolarization.^10^ It is rapid kindling of neurons induced by repeated pulses of current that eventually results in seizure.^11^ Since the properties of electric current entering the brain are within physiological parameters, and the temperature rise in the intracranial tissues is negligible, medical complications of ECT attributed to the current per se are very few, such as electric current induced bradycardia. They mainly arise from three factors: convulsions, hemodynamic alterations, and effects of anesthesia.^6^ Even when adverse effects occur, attribution to ECT is confounded by other medical factors.^12^

## MATERIALS AND METHODS

We conducted the literature search using the following keywords: “Electroconvulsive therapy OR ECT,” “contraindications,” and “medical precautions.” Additionally, we performed searches for individual systems and conditions, such as “cardiovascular,” “aneurysms,” “arrhythmias,” “myocardial infarction,” “cardiac failure,” “aortic stenosis,” “deep vein thrombosis,” “DVT,” “aortic aneurysm,” “stroke,” “intracranial space‐occupying lesions,” “delirium,” “dementia,” “skull defects,” “myasthenia gravis,” “myopathies,” “fractures,” “pacemakers,” “implant,” “pregnancy,” “epilepsy,” “medications,” “kidney diseases,” “pheochromocytoma,” “intraocular pressure,” “obesity,” “infection,” “COPD,” and “COVID 19.” The search was limited to English‐language and human studies.

To reduce the risk of selection bias, we reviewed previous reviews and practice guidelines on ECT administration in the presence of medical conditions.^13^, ^14^, ^15^, ^16^, ^17^ Using the OVID search tool, we searched for each condition in PubMed and EMBASE till March 2024. All abstracts were screened and, when relevant, two independent reviewers evaluated the full texts. Discussions with a third reviewer resolved disparities. Due to significant heterogenicity of the topic, we adopted the state‐of‐the‐art (SOTA) review. A SOTA review is a type of literature review that emphasizes the most recent and innovative developments in a specific field or research area, such as “the application of ECT in complex medical conditions.” Although we conducted a comprehensive and systematic search, our primary focus remained on recent advancements, emerging trends, and cutting‐edge findings, ensuring alignment with the principles and purpose of a SOTA review. This approach allowed us to capture the forefront of current knowledge and highlight key innovations within the field.

**Figure 1:**
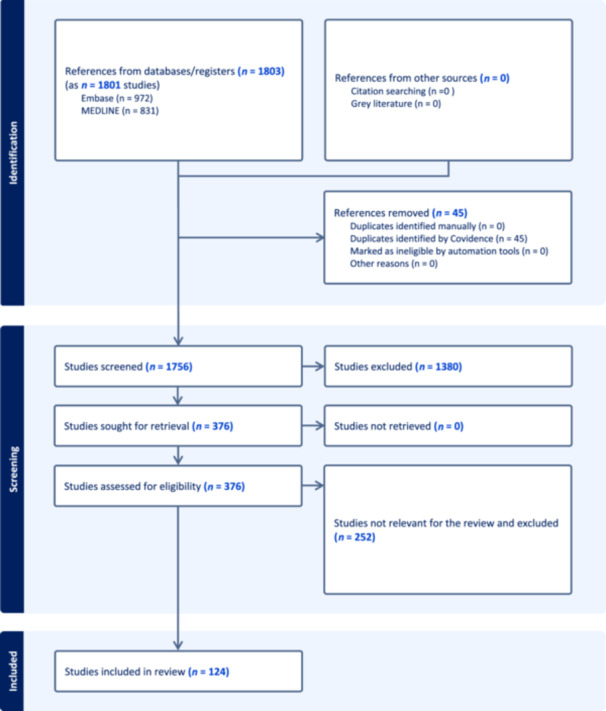
PRISMA flow diagram: electroconvulsive therapy (ECT) application in medically compromised patients.

Using the COVIDENCE tool, we initially identified 1803 studies. A large proportion of these were excluded in the initial screening due to being opinion pieces, commentaries, unstructured reviews, conference presentations, or unstructured case studies. After this preliminary filtration, 376 studies remained for full‐text review. Ultimately, only 124 papers were found to be relevant for this review (Figure 1).

## RESULTS

### Cardiovascular diseases

ECT was given in almost all cardiac conditions.^16^ Most of the cardiovascular complications associated with ECT are minor and manageable.^17^ The electrical stimulus is parasympathetic, causing transient asystole and bradycardia lasting for several seconds, followed by sympathetic activity resulting from seizure.^18^ The catecholamine surge may persist for minutes, translating into various hemodynamic changes, namely, tachycardia, hypertension, and increased cardiac contractility.^19^ BP may rise twofold, and heart rate by 1.45 times.^5^ Enhanced cardiac contractility may increase mean cardiac output up to 81%.^4^ All these changes can be effectively blocked by β‐adrenoceptor antagonists.^5^ With bifrontal stimulation, sympathetic stimulation occurs without a preceding phase of significant bradycardia (Table 1).^18^, ^20^

(a) Arrhythmias
  The reported incidence varied from 8% in healthy people to 80% in patients with pre‐existing cardiac diseases.^21^, ^22^ The most common complication is ventricular ectopic.^22^ More serious arrhythmias reported from one study of 2279 patients who were given 17,394 ECTs included second degree AV block (*n* = 2), bradycardia (*n* = 2), ventricular tachycardia (*n* = 2), ventricular fibrillation (*n* = 1), prolonged asystole, and cardiac arrest (*n* = 1).^12^ Notably, no one died during or soon after ECT. Another review of 80 patients showed ECT was relatively safe in older patients with pre‐existing cardiac risk factors.^36^ Persistent ECG changes or an elevation of cardiac enzymes were not observed after ECT.^37^, ^38^
(b) Myocardial infarction
  Fatality in the minutes after ECT was documented in relatively old literature in a person who had an autopsy‐proven myocardial infarction.^23^ Left ventricular hypertrophy was present in this case. There is a risk of cardiac arrhythmias, recurrent infarction, and myocardial rupture with ECT, particularly in the first 10 days after infarction. This risk is deemed to be reduced after 3 months.^15^ However, ECT was safely given without dose titration for a patient with catatonia 4 weeks after myocardial infarction using a β‐adrenergic antagonist and close monitoring of cardiac parameters.^24^
(c) Cardiac failure
  In a case series, ECT was not associated with any sentinel event in five patients with congestive cardiac failure.^15^ Published data support the safety of ECT with cardiac ejection fraction as low as 15%, including in older individuals.^25^ In such instances, ECT was administered with medications that reduce cardiac workload (e.g., lisinopril) or β‐blockers that decrease tachycardia and hypertension.
(d) Aortic stenosis
  A case series of 10 patients and a single case report demonstrated the safe administration of ECT in severe aortic stenosis.^39^, ^40^ Two patients had low BP 1 min after ECT, but this was successfully managed.
(e) Deep vein thrombosis
  ECT is an effective treatment for mood disorders and catatonia. These conditions can be associated with diminished mobility and deep vein thrombosis (DVT).^41^ Pulmonary embolism (PE) due to dislodgement of the thrombus during convulsion is a known theoretical risk in the presence of DVT. In line with this, there are documented instances of PE developing after ECT, including fatal ones.^26^, ^27^, ^28^, ^29^ However, in several patients with DVT, ECT was safely administered.^41^, ^42^ The outcomes of ECT in the presence of DVT are thus mixed and form an area that warrants further investigations, given the lethal nature of PE. A case report showed sudden death after ECT, which was possibly linked to DVT in the context of COVID‐19 infection, but without a definite conclusion.^43^ Possible contributing factors to embolization in DVT include the above‐knee location and size of the thrombus. The commencement of anticoagulants does not offer absolute protection from PE due to an already‐developed venous thrombus, which may still dislodge during convulsive movements. If the clinical indication for ECT is compelling, discussion with vascular surgeons and anesthetists is imperative to achieve complete muscle relaxation and optimal outcomes.
(f) Aortic aneurysm
  A case series of eight patients suggested that ECT was safe in people with aortic aneurysms with aortic diameters ranging from 3.3 to 5.3 cm.^44^

**Table 1:** ECT in the presence of cardiovascular and neurological conditions.

Medical conditions	Level of evidence	Adverse events	Death	Caution/comments
Cardiac arrythmias^12^, ^21^, ^22^	Retrospective chart review	Ventricular ectopic most common, ventricular tachycardia, ventricular fibrillation extremely rare	None	Cardiac evaluation and monitoring, including review of electrolytes such as potassium and magnesium
	Case reports			
Myocardial infarction^15^, ^23^, ^24^	Case reports	Arrythmias	Yes	High degree of caution
				Wait for 3 months. Appropriate use of β‐blockers
				Avoid titration or consider bifrontal placement
Cardiac failure^15^, ^25^	Case series	Hypertension	No	Cardiac evaluation
	Chart reviews			Angiotensin system antihypertensives
Deep vein thrombosis^26^, ^27^, ^28^, ^29^	Case reports	Pulmonary embolism	Yes	High degree of caution
	Reviews			Hematology consultation
				Anticoagulation
				Extended monitoring
				Maximum muscle paralysis with an increased dose of muscle relaxants
Intracranial aneurysms^30^, ^31^, ^32^	Case reports	Headache	Yes	Neurosurgery evaluation
	Reviews			High degree of caution for aneurysms more than 1.5 cm
				Esmolol with intraarterial blood pressure monitoring
Brain tumors^33^, ^34^, ^35^	Case reports	Secondary myoclonic seizure, ping‐pong gaze, Todd's paralysis, delirium and coma	Not in the recent literature	Moderate degree of caution
	Reviews			Neurosurgery opinion
				Dexamethasone
				Phenytoin

#### Risk mitigation in the presence of cardiovascular diseases

A waiting period of 3 months after myocardial infarction is regarded as safe for ECT.^15^ Cardiovascular complications can be mitigated by avoiding multiple stimulations and using β‐blockers to attenuate hypersympathetic response in high‐risk populations. Patients should be advised to take these medications the morning before treatment with sips of water.^16^ Dose titration may be avoided, or bifrontal placement may be considered for patients at risk of asystole, such as those taking β‐blockers.^25^ After successfully treating patients with cardiac failure with an ejection fraction varying from 20% to 25%, Stern and colleagues developed a protocol that included administering regular cardiac medications 60–90 min before ECT and avoiding anticholinergic drugs.^45^

#### Cardiac monitoring during ECT

Given the significant hemodynamic alterations, in particular seizure‐related catecholamine surge, cardiac monitoring is an essential part of ECT. The monitoring normally entails electrocardiography, pulse oximetry, and noninvasive intermittent BP measurements.^46^ This level of monitoring does not capture rapid hemodynamic changes. Novel noninvasive devices, such as Nexfin HD, can continually monitor cardiac output and beat‐to‐beat arterial BP. Since the hemodynamic changes associated with ECT are short‐lived, techniques that use continuous monitoring to detect rapid changes can be useful, especially in patients with high‐risk conditions. Patients with low cardiac ejection fraction will require monitoring of BP concurrently with both an arterial line and dual BP cuff monitoring using monitors on both arms with continuous BP cycling.^47^ Esmolol, a β‐adrenergic blocker, attenuated an increase in heart rate and systolic, diastolic, and mean arterial BP from a dosage of 1.0 mg kg^−1^ onward.^48^

### Neurological and neurosurgical conditions

(a) Aneurysm and stroke
  So far, 27 cases of ECT in patients with aneurysms have been published.^30^ ECT was safe with secured and unsecured aneurysms up to a size of 2.1 cm, except in one instance where an 84‐year‐old patient died from subarachnoid hemorrhage following rupture of an anterior cerebral artery aneurysm of 1.9 cm 2 days after 10th right unilateral ECT.^31^, ^32^ The patient received esmolol; the peri‐ictal BP increased to 207/89 mm Hg. This report suggests that ECT is not universally safe in patients with aneurysms. The risk appears to increase with large aneurysms. One case report demonstrated the safety of ECT within 2 weeks after stroke.^49^ Another comparative study showed no difference in the delirium incidence between ECT given after stroke and without stroke; all cases of post‐ECT delirium during the poststroke period were seen after caudate stroke.^50^
(b) Intracranial space‐occupying lesions
  An earlier review of reports published until 1984 showed complications in 74% (*n* = 38) and mortality in 8% (*n* = 29) of patients with intracranial neoplasms after ECT.^33^ Complications included aphasia, ataxia, hemiplegia, delirium, and coma. Although the authors suggested that ECT could have precipitated deaths in 8%, some of them had aggressive tumors like glioblastoma. A more modern systematic review and retrospective data found that ECT was largely safe, with no mortality in patients with intracranial tumors.^34^, ^35^ Buday et al. reviewed 40 reports published until 2019. Reversible adverse reactions reported in six patients (15%) were secondary myoclonic seizure, ping‐pong gaze, Todd's paralysis, delirium, and coma. Differences between the earlier review and Buday et al. were prior knowledge of tumors, more benign tumors, dose titration, and use of dexamethasone in the latter.^33^, ^34^ The largest reported tumor was 8.1 × 7.6 × 4.1 cm, and ECT was not associated with adverse effects in this case.^51^ A mild mass effect does not appear to preclude ECT if there is no midline shift.^52^ Case reports suggest that ECT is safe in idiopathic intracranial tension.^53^
(c) Delirium and dementia
  Although ECT may cause transient postictal confusion, there is no evidence to suggest that delirium and dementia are contraindications for ECT. The evidence indicates that ECT is beneficial in reducing behavioral symptoms in a subgroup of patients with these conditions.^54^, ^55^
(d) Skull pathology
  Skull defects can cause the direct entry of electricity into the brain with severe consequences. Given its high impedance, the skull attenuates the current flow so that only a small portion enters the brain.^9^ Patients may be regularly screened for skull deficits because of trauma or surgery and lytic skull lesions.

#### Risk mitigation

A retrospective study identified one abnormal scan that prevented ECT out of 108 scans (0.93%) performed as routine pre‐ECT imaging.^56^ Considering such a low probability and the relative safety of ECT, brain imaging may be performed for patients at risk, for instance those with prior aneurysms, heritable disorders associated with intracranial aneurysms, neurological symptoms such as headaches, and those above 60 years of age with a history of hypertension or smoking. Neurology and cardiology consultations are required in such situations. While esmolol is administered, intra‐arterial BP monitoring at the first session of ECT to understand the exact real‐time variation of BP during the preictal, ictal, and postictal phases is required to determine sufficient esmolol dose for subsequent sessions. Risk management in the presence of intracranial tumors includes the administration of dexamethasone to reduce edema around the tumor and phenytoin to reduce the risk of recurrent seizures.^34^ An interdisciplinary approach encompassing consultations with neurologists, neurosurgeons, and anesthetists may reduce the risk of complications (Table 1).

### Neuromuscular disorders

1. Myasthenia gravis
  Myasthenia gravis (MG) is an autoimmune disorder characterized by antibodies against nicotinic acetylcholine receptors (anti‐AChAb), affecting the neuromuscular junction. Case reports suggest ECT is safe in patients with MG, provided adequate precautions are taken.^57^, ^58^ During the active phase of the disease, ECT should be avoided, and the patient should be in reasonable remission before undergoing ECT. The patient's condition can be managed through expert opinion from a neurologist and regular monitoring of pulmonary function tests, plasma pseudocholinesterase levels, and anti‐AChAb levels.^58^ Anticholinergic agents are usually avoided to prevent a cholinergic crisis. Although suxamethonium was safe during remission, response to succinylcholine during ECT was variable, and in one instance, rocuronium was used with sugammadex reversal.^57^, ^59^ A significant reduction in endplate receptors at the neuromuscular junction may limit the action of succinylcholine, causing resistance or decreasing the safety margin.^60^ Prednisolone and cholinesterase inhibitors, commonly used in MG, can prolong the half‐life of succinylcholine and result in longer‐than‐usual neuromuscular blockade.^60^, ^61^ Cholinesterase inhibitors can increase the risk of asystole and bradycardia, and in these situations, bifrontal ECT and stimulation without titration may be considered.^62^
2. Inflammatory myopathies
  In patients with inflammatory myopathy, neuromuscular blockers require caution due to potential complications such as delayed or prolonged effects, hyperkalemia, diaphragmatic dysfunction, impaired coughing, and swallowing difficulties requiring special anesthetic care. These concerns arise from a rise in serum creatine phosphokinase (CK) after ECT.^37^, ^63^ Empirical evidence also suggests that CK levels normalized with subsequent ECT when administered with succinylcholine without worsening muscle disease.^37^, ^63^, ^64^ Ntatsaki et al. used the nondepolarizing muscle relaxant mivacurium 0.2 mg/kg followed by reversal with neostigmine and glycopyrrolate in a patient with inclusion myositis.^65^ Management strategies in inflammatory myopathies include monitoring CK levels and providing enhanced anesthetic care to prevent aspiration.

### Bone fractures, muscle tears, and vascular injuries

In the preanesthetic era of ECT, musculoskeletal injuries were expected during convulsive movements. In modern practice, fractures are not necessarily contraindications for ECT. Case series suggest that ECT can be safely and successfully given to patients with bone fractures with coordinated care from orthopedic surgeons and anesthetists.^66^, ^67^ Similarly, ECT was safely administered 2 weeks after the repair of the severed strap muscle and the anterior jugular vein.^68^ Precaution is required in such conditions; succinylcholine in an adequate dose (1.5 mg/kg) may bring sufficient muscle relaxation, protecting musculoskeletal structures. There were cases of successful ECT with no adverse events in patients with Harrington rod implants.^69^

### Implanted devices

(a) Pacemakers
  Pacemakers or implanted defibrillators are not contraindications for ECT.^70^, ^71^, ^72^ Supraventricular tachycardia was reported in one patient, which resolved promptly and did not prevent further ECT. The synchronous mode is preserved as serious arrhythmias are a risk of the asynchronous mode. A cardiologist's opinion must be sought before proceeding to ECT for patients with these devices.
(b) Deep brain stimulators
  A review of 21 cases showed that ECT was safe with deep brain stimulators, provided precautions were taken.^73^ Burr holes through which deep brain simulators (DBSs) were placed must be avoided during ECT, and the device is to be reprogrammed to 0 V and switched off. Although the DBS was turned off during the whole ECT course in seven instances, it was only turned off immediately before ECT in other cases and turned on in recovery units. There was no evidence of brain tissue damage, a change in neurological function, DBS device damage, or reprogramming because of ECT. In studies where DBS electrode placement was confirmed before and after ECT by neuroimaging, there was no change in the position of the DBS electrodes.
(c) Cochlear implant
  A cochlear implant was previously considered an absolute contraindication for ECT.^74^ As years passed, it was found that cochlear implant users needed ECT. Reflecting on this need, the Danish Psychiatric Association and other authors reviewed the literature and later concluded that a cochlear implant is not an absolute contraindication.^75^ ECT did not damage the implant even with the highest level of energy (1004 mC) delivered from an ECT device.^75^ Stimulations in all but one patient were administered through the side contralateral to the implant; for one patient who had the implant fitted to the right ear, right unilateral ECT was administered.^76^ Discomfort around the jaw was the only reported adverse effect. However, given the dearth of data about ipsilateral ECT with the cochlear implant, it is safe to administer stimulations at the maximum distance away from the implant. Bifrontal ECT is safe in this regard.

### Pregnancy

The official joint statement from the American Psychiatric Association and the American College of Obstetricians and Gynecologists, several original studies, and meta‐reviews indicate that ECT is usually a safe and effective treatment in pregnancy.^77^, ^78^, ^79^ However, some authors published a contradictory report and cautioned that ECT should be used as a last resort in pregnancy.^80^ The findings included a reduced fetal heart rate and several fetal deaths (7.1% from ECT given to 169 pregnant women).^80^ In one instance, fetal death occurred after status epilepticus in the mother following ECT.^81^ Other adverse events were fetal arrhythmias, premature birth, preeclampsia, vaginal bleeding, and uterine contractions without labor. Although there are inconsistent views, most reviews are reassuring, and reports differ due to varied attributions of adverse events to ECT.^77^, ^78^, ^79^ A recent review rated complications as low‐to‐moderate grade and not life‐threatening to pregnant women.^82^ Joint management with the obstetric team involving frequent fetal monitoring is critical. A semi‐prone position, endotracheal intubation to reduce the risk of aspiration, the elevation of the right hip to improve the placental perfusion, extended fetal monitoring, and inhalational anesthetics to minimize the risk of uterine contractions are essential considerations for ECT in pregnancy.^83^ There are few reports on ECT during special situations like twin pregnancies where there were no adverse outcomes.^84^, ^85^

### Epilepsy

ECT has an anticonvulsant effect and is hence not contraindicated in epilepsy or if there is a history of previous seizures.^86^ However, a neurological evaluation is to be completed to determine the cause of the seizure, which may imply extra caution for ECT.

### ECT and concomitant medications

(a) Theophylline
  Co‐administration of ECT and theophylline medications was followed by prolonged and recurrent seizures in one patient and prolonged seizure (190 s) in another.^87^, ^88^ Theophylline is known to induce seizure at a serum level of 25–50 μg/mL. However, recurrent seizure after ECT occurred at 22.6 μg/dL, measured 3 h after ECT, suggesting an interaction.^87^ It is therefore crucial to measure the theophylline level before ECT and ensure it is well below seizure‐prone levels. In most documented cases, ECT was uneventful when administered with theophylline.^89^ Interestingly, theophylline with a level of 10–15 μg/mL was also used to induce or generate sufficiently long seizures for therapeutic purposes.^89^
(b) Lithium
  Earlier case reports and retrospective chart reviews showed prolonged seizure, apnea, and delirium with concomitant lithium and ECT.^90^, ^91^, ^92^ However, a recent prospective randomized controlled trial and a retrospective study did not observe severe complications because of this combination when lithium levels were below 0.6 mEq/L.^93^, ^94^ Caution is still required, and lithium must be withheld before ECT in the morning.
(c) Antiepileptics
  Contrary to the conventional wisdom, several reports including controlled trials showed that antiepileptic medications did not affect seizure quality or the clinical efficacy of ECT.^95^, ^96^, ^97^ Therefore, these medications may be continued during ECT. Although results of combined ECT and benzodiazepines are mixed, the overall picture suggests that seizure can be achieved with clinical efficacy, especially in catatonia, for which this combination is highly useful.^97^ Stimulus dose titration to determine the seizure threshold is recommended when ECT is given with antiepileptics (Table 2).

**Table 2:** ECT in other medical conditions.

Conditions	Level of evidence	Adverse events	Death	Caution/comments
Musculoskeletal injuries^66^, ^67^	Case reports	None reported	No	Orthopedic opinion
				Additional anesthetic consultation
				Succinyl choline
				1.5 mg/kg
Vascular injuries^68^	Single case report	None reported	No	Vascular surgery opinion
				Additional anesthetic consultation
				Succinyl choline
				1.5 mg/kg
Pacemakers Defibrillators^70^, ^71^, ^72^	Case series	SVT	No	Keep synchronized mode
				Cardiac evaluation
Deep brain stimulator^73^	Case reports	No	No	Avoid burr holes.
				Reprogram to 0 during ECT
Cochlear implant^74^, ^75^	Case reports	No	No	Electrodes farther from the implantContralateral stimulation or bifrontal stimulation
Medications				
Theophylline^87^, ^88^	Case report	Status epilepticus prolonged seizure	No	Extra monitoring
				Review theophylline level
Lithium^90^, ^91^, ^92^, ^93^, ^94^	Chart reviews	Delirium, prolonged apnea	No	Aim low serum lithium below 0.6 mEq/L
Anticonvulsants^95^, ^96^, ^97^	RCT	Prolonged seizure	No	Avoid lithium 24 h prior to ECT
	RCT			Dose titration to determine threshold
Opthalmic Disease^98^, ^99^, ^100^, ^101^, ^102^, ^103^	Case reports	Increased IOP, vitreous detachment	None	Moderate caution, check IOPRetinal detachment reported after ECT
Obesity^104^, ^105^	Case series	Desaturation	No	Supine position with a 15–30° elevation of the upper body
				Supraglottic device and noninvasive positive pressure ventilation
				Non‐depolarizing agents

Abbreviations: ECT, electroconvulsive therapy; IOP, intraocular pressure; RCT, randomized controlled trial; SVT, supraventricular tachycardia.

### Other conditions

(a) Kidney diseases and pheochromocytoma
  Individuals with renal failure undergoing hemodialysis received ECT without adverse reactions.^106^, ^107^, ^108^ Caution is required to reduce BP before administering ECT with β‐blockers such as esmolol.^107^ Pheochromocytoma is a rare neoplasm with an incidence of 0.2–0.8 per 100,000 individuals.^109^ Hypertension can occur in this condition because of the catecholamine‐secreting tumor. A 34‐year‐old woman with pheochromocytoma died 3 h after ECT due to sudden vascular collapse.^110^ The authors of this report proposed that death could be attributed to ECT, vascular collapse from sodium thiopental, or emotional stress. Notably, the pulse rate was 100/min, and there was no record of hypertension. In another instance, a 27‐year‐old man with pheochromocytoma received ECT. In minutes after the first stimulation, the systolic BP went up to 260 mm Hg, which was reduced by administering a β‐blocker. The patient had subsequent ECT without complications.^111^
(b) Ophthalmic conditions
  An increase in intraocular pressure (IOP) occurs immediately after the beginning of the seizure, which returns to normal ∼3 min after stimulation.^98^ While retinal detachment was reported during the recovery from bilateral ECT in one instance, no adverse ophthalmological outcomes were noted after ECT in patients who had previous retinal detachment, albeit 30 years ago in one case.^99^, ^100^, ^107^ No ocular complications occurred in relation to ECT after cataract surgery, in glaucomatous eyes, or in the presence of titanium‐based trabecular microbypass stents for open‐angle glaucoma treatment.^102^, ^103^, ^112^ There is a need for regular ophthalmic consultation and IOP measurements when a patient presents with such risk factors.
(c) Respiratory diseases
  Considering that ECT involves general anesthesia and assisted ventilation, careful consideration of pulmonary conditions is crucial. According to one retrospective study, four of 34 patients with asthma experienced an exacerbation of symptoms, which were successfully managed by standard anti‐asthma treatments.^113^ However, no complications were reported after ECT in patients with chronic obstructive pulmonary disease.^114^ As mentioned earlier in this review, the use of theophylline should be carefully evaluated.
(d) Fever and infection
  A single case report showed the safety and efficacy of ECT in a 4‐year‐old boy with febrile infection‐related epilepsy syndrome (FIRES) that followed pharyngitis and high fever.^115^ ECT was successfully given to patients with symptomatic COVID‐19.^116^ In the Regestein and Reich case series, three out of 19 patients who received ECT had signs of infection, including Gram‐positive septicemia and unrelenting fever.^15^ Tachycardia was observed, with the highest reported pulse rate of 180/min. Death was reported in a young patient with massive PE, pleural effusion, and staphylococcal pneumonia after developing ventricular fibrillation during ECT.^15^
(e) Obesity and conditions that increase the risk of aspiration
  Obesity can pose challenges during ECT, given delayed gastric and esophageal emptying and difficulties in protecting the airway.^104^, ^117^ Also, prospective observational data suggest that obesity and the duration of seizure were the factors correlated with post‐ECT desaturation.^118^ However, the duration of unconsciousness during ECT is relatively brief. A review of 50 obese patients with a body mass index (BMI) of 45 or above who received 660 ECT sessions demonstrated a safe administration of the treatment without a requirement for tracheal intubation.^104^ Patients fasted overnight and received 100% oxygen with assistance of positive pressure using a face mask. ECT was performed in the supine position with a 15–30° elevation of the upper body. In another instance, when hypoxic episodes occurred because of suspected inadequate ventilation, a lower dose of succinylcholine, 0.5 mg/kg based on the total body weight, helped to restore adequate oxygenation.^119^ This may be understood against a background of increased muscular consumption of oxygen because of succinylcholine‐induced fasciculations.^120^ Some authors, therefore, recommend nondepolarizing agents, such as rocuronium, in obesity.^105^ Other strategies to reduce the risk of adverse outcomes with ECT in obesity include a supraglottic device and noninvasive positive pressure ventilation.

### Age

Although advanced age is associated with increased medical morbidities, old age per se is not associated with mortality after ECT.^121^ Until now, the oldest person to receive ECT was a 100‐year‐old woman who safely underwent the treatment.^40^ ECT is safe in children.^122^, ^123^ No evidence supports the fear that ECT adversely affects brain growth (Table 3).

**Table 3:** ECT in special physiological conditions.

Conditions	Level of evidence	Adverse events	Deaths	Comments
Extremes of age^121^, ^122^	Systematic review	None	None	Electroconvulsive therapy is safe in extremes of age
	Care reports			
Pregnancy^77^, ^79^, ^80^, ^82^, ^83^	Systematic reviews	Fetal arrythmias, premature birth, preclampsia, vaginal bleeding, and uterine contractions without labor	Yes	Obstetric evaluation
			(fetal deaths)	High degree of caution
				Semiprone position, endotracheal intubation to reduce the risk of aspiration
				Elevation of the right hip to improve the placental perfusion
				Fetal monitoring, and inhalational anesthetics to minimize the risk of uterine contractions

## LIMITATIONS

The above results are to be cautiously interpreted. They are from limited evidence constituted by case reports and case series, uncontrolled data, and retrospective reviews. The reproducibility of some of these results is thus limited. Also, this report is prepared based on extant literature in which serious and fatal outcomes may not be reported. Still, ECT is a time‐tested intervention in psychiatry and has remained in practice for over eight decades. This narrative review is biased toward a nonsystematic selection of the literature without a specific protocol and hence is not inclusive of every report of ECT in one or another medical condition.

## CONCLUSION

In general, ECT can be safely administered to patients with various medical conditions and physiological complexities. In certain conditions, ECT may be avoided or administered with high caution if indicated based on a favorable risk–benefit ratio as relevant for individual patients. These conditions include a recent myocardial infarction in the last 4 weeks, intracranial aneurysms of more than 1 cm in size, proximal DVT, pheochromocytoma, and systemic infections. In particular, fatalities associated with DVT warrant extreme precaution and monitoring in this condition. In a nutshell, the extension of ECT to patients with medical and physiological complexities expands the scope of this treatment and thus the horizon of psychiatry.

## AUTHOR CONTRIBUTIONS

Alby Elias, Soumitra Das, Sarabjit Loyal, and Naveen Thomas contributed to the conceptualization and design of the study. Data acquisition and analysis were performed by Alby Elias, Soumitra Das, and James Kirkland. Naveen Thomas, Sarabjit Loyal, and Soumitra Das contributed significantly to drafting the manuscript and developing the figures and tables.

## CONFLICT OF INTEREST STATEMENT

The authors declare no conflicts of interest.

## ETHICS APPROVAL STATEMENT

The ethics approval statement is not applicable.

## PATIENT CONSENT STATEMENT

The patient consent statement is not applicable.

## CLINICAL TRIAL REGISTRATION

The clinical trial registration is not applicable.

## Data Availability

All relevant data are within the paper.
